# Neuroscience in Asia-Pacific : Horizons and Innovations

**DOI:** 10.1016/j.ibneur.2025.08.022

**Published:** 2025-08-26

**Authors:** Wael Mohamed, Toshihide Yamashita, Rajat Sandhir, Roongroj Bhidayasiri

**Affiliations:** Basic Medical Science Department, Kulliyyah of Medicine, International Islamic University Malaysia (IIUM), Kuantan, Pahang, Malaysia; Department of Molecular Neuroscience, Graduate School of Medicine, The University of Osaka, Suita, Osaka, Japan; Department of Biochemistry, Hargobind Khorna Block, Panjab University, Chandigarh 160014, India; Chulalongkorn Centre of Excellence for Parkinson’s Disease & Related Disorders, Department of Medicine, Faculty of Medicine, Chulalongkorn University and King Chulalongkorn Memorial Hospital, Thai Red Cross Society, Bangkok 10330, Thailand; The Academy of Science, The Royal Society of Thailand, Bangkok 10330, Thailand

In recent years, there has been an increasing worldwide acknowledgement of the significance of brain research, shown by the initiation of prominent brain projects in Europe, and the United States. The Asia-Pacific area has traditionally been under-represented in this domain; nevertheless, significant advancements are now evident, as shown by the China Brain Project (Poo et al., 2016) and the Japan Brain Project (Okano et al., 2015).

Neuroscience in the Asia-Pacific region has witnessed remarkable growth over the past decades, emerging as a significant contributor to global scientific progress. The region’s vast diversity—spanning populations with distinct genetic profiles, varied environmental exposures, and unique cultural contexts—provides a fertile ground for exploring the complexities of brain function and neurological disorders. Research institutions and universities across Asia-Pacific are increasingly recognized for their excellence, producing impactful studies that address both region-specific and universal challenges in neuroscience. Collaborative efforts within and beyond national borders have further amplified the region’s influence, enabling the integration of cutting-edge methodologies, advanced neurotechnologies, and translational approaches that bring laboratory discoveries closer to clinical application.

The International Brain Research Organization (IBRO) plays a pivotal role in this advancement. Established with the mission to promote and support neuroscience research and education worldwide, IBRO aims to foster collaboration, build capacity, and facilitate knowledge exchange among neuroscientists across all regions. Its vision is to create an inclusive global neuroscience community that drives discovery, innovation, and equitable progress in understanding the brain. IBRO’s initiatives, including training programs, research funding, and networking opportunities, have been instrumental in empowering scientists in the Asia-Pacific and ensuring that their work reaches international visibility. In the last three years (2023–2025), IBRO has markedly progressed neuroscience in the Asia-Pacific area with focused initiatives in training, research financing, and networking. Diversity Grants (2024) facilitated inclusive events, promoting enhanced gender and regional representation. The IBRO Associate School on Cerebral Organoid Models (2024, UPM Malaysia) offered comprehensive lectures, laboratory training, and ethical discourse, preparing emerging scientists with advanced competencies in neurodegenerative research. Networking possibilities, shown by the Early and Mid-Career Researcher Social (2024), enhanced mentoring, cooperation, and exposure within the area neuroscience community. In 2025, Neuroscience Training Grants facilitated early-career researchers' participation in advanced courses, and Collaborative Research Grants supported worldwide collaborations and capacity enhancement across labs. These programs have together strengthened Asia-Pacific neuroscientists by broadening access to skills, promoting inclusion, and augmenting the worldwide exposure and influence of their research. As part of this mission, IBRO Neuroscience Reports serves as an open-access, peer-reviewed platform dedicated to publishing high-quality research across the full spectrum of neuroscience. The journal seeks to promote global representation in neuroscience literature, highlight diverse perspectives, and accelerate the dissemination of knowledge to both the scientific community and the broader public.

This special issue of IBRO Neuroscience Reports presents a rich compilation of peer-reviewed studies led by Asia-Pacific researchers, reflecting the region’s growing leadership in both basic and clinical neuroscience. The articles span a wide range of topics and methodologies, offering insights into cellular, molecular, behavioral, and clinical aspects of brain science. One study reveals that gliogenesis (Chen et al., 2025), but not neurogenesis, predominates during the acute phase of vestibular compensation after unilateral vestibular neurectomy, shedding light on the cellular mechanisms underlying balance recovery. Another study by Kamboj et al. (2025) demonstrates that N-acetyl-L-cysteine mitigates diabetes-induced impairments in the sciatic nerve, identifying a promising therapeutic approach for peripheral neuropathy. Behavioral, neurochemical, and histological evaluations in a reserpine-induced fibromyalgia mouse model provide critical comparisons between two dosing regimens, advancing preclinical pain research (AboTaleb et al., 2024). In regenerative neuroscience, an innovative investigation (Kim et al., 2024) shows that pericyte derivation and transplantation can restore the blood–CNS barrier, offering hope for targeted therapies in neurological disorders. Motion sickness research (Zhang et al., 2023) is enriched by a study evaluating rodent proxies to improve translational validity in anti-nausea treatment development. Clinically, an interventional reperfusion strategy for acute embolism of the middle cerebral artery trunk in the context of contralateral internal carotid artery congenital absence addresses treatment challenges in rare vascular anatomies (Wang et al., 2023). A comprehensive review of artery of Percheron infarction offers diagnostic and therapeutic guidance for this rare stroke variant (Li et al., 2023). In psychiatry, a critical synthesis of pre- and post-treatment symptomatic and morphological changes in schizophrenia bridges clinical findings with neuroimaging evidence (Chatterjee and Chatterjee, 2023). A targeted review on neurodegeneration–inflammation crosstalk identifies therapeutic opportunities for a range of neurological diseases (Mohamed et al., 2023). Molecular virology research reveals that pyruvate dehydrogenase kinase 1 promotes neuronal apoptosis during Japanese encephalitis virus infection, suggesting novel intervention targets (Chakraborty et al., 2022). A forward-looking perspective on integrating nutriepigenomics into Parkinson’s disease management highlights the potential of dietary epigenetic modulation in the omics era (Razali et al., 2022). Finally, mapping mGluR1α expression in the hippocampus, subiculum, entorhinal cortex, and superior temporal gyrus in Alzheimer’s disease provides crucial insights into receptor-specific alterations in disease progression (Yeung et al., 2022).

The significance of this special issue lies not only in its scientific content but also in its role as a milestone in the progression of neuroscience in the Asia-Pacific. By bringing together such diverse and high-caliber studies, this collection amplifies the visibility of regional research, fosters cross-disciplinary dialogue, and strengthens the global integration of Asia-Pacific neuroscience. It stands as a testament to the innovation, collaboration, and dedication of researchers in the region, reinforcing IBRO’s mission to advance brain science for the benefit of all. Through the dissemination of these findings, we hope to inspire further breakthroughs, nurture future leaders, and solidify the Asia-Pacific’s role as a driving force in the global neuroscience community.

The Asia-Pacific area is leading revolutionary advancements in neuroscience, using its diversified demographics, distinct disease loads, and fast expanding research infrastructure. To fully actualise this potential, it is imperative to prioritise cross-border partnerships, standardise data-sharing systems, and engage in capacity-building efforts for emerging scientists and clinicians. We advocate for the creation of more robust regional networks and collaborations that connect academics, industry, and policy. By coordinating resources and goals, the Asia-Pacific region can establish itself as a worldwide leader in neuroscience innovation, converting findings into concrete health advantages for its varied people and beyond.
